# Supplementary material for: Chromosomal‐level reference genome of the incense tree Aquilaria sinensis
Source: Mol Ecol Resour. 2020 Apr 8;20(4):971–9. doi: 10.1111/1755-0998.13154 (PMC7496549; doi:10.1111/1755-0998.13154)
Supplement: Supplementary file 1 — TablesS1‐S6 [file MEN-20-971-s001.docx]

**Supplementary Information: Chromosomal-level reference genome of the incense tree *Aquilaria sinensis***

Wenyan Nong^1,^^, Sean T.S. Law^1,^^, Annette Y.P. Wong^1^, Tobias Baril^2^, Thomas Swale^3^, Lee Man Chu^4^, Alexander Hayward^2^, David T.W. Lau^5^, Jerome H.L. Hui^1,*^

1. School of Life Sciences, Simon F.S. Li Marine Science Laboratory, State Key Laboratory of Agrobiotechnology, The Chinese University of Hong Kong, Hong Kong

2. Department of Conservation and Ecology, Penryn Campus, University of Exeter, United Kingdom

3. Dovetail Genomics, United States of America

4. School of Life Sciences, The Chinese University of Hong Kong, Hong Kong

5. Shiu-Ying Hu Herbarium, School of Life Sciences, The Chinese University of Hong Kong, Hong Kong

**Table of Contents**

**Supplementary information S1.** Summary of genomic and transcriptomic data generated in this study………………………………………………………………………………….... P. 3

**Supplementary information S2.** Statistics of the eight assembled pseudomolecules……. P. 4

**Supplementary information S3.** Summary of repeat types present in the genome of the incense tree………………………………………………………………………………… P. 5

**Supplementary information S4.** Summary of repeat types present in the genomes of the incense tree and other members of the Malvales for which genome assemblies exist….….. P. 6

**Supplementary information S5.** Gene sequences and locations of genes identified in Figure 4………………………………………………………………………………...………… P.7-9

**Supplementary information S6.** A) Neighbour-joining tree of sesquiterpene synthases; B) Maximum-likelihood tree of sesquiterpene synthases…………………………...…….. P. 10-11

**Supplementary information S1.**

**Supplementary information S2.**

| Sum = 783,887,358, n = 14276, average = 54,909.45, largest = 114,924,005 | | | | |
| --- | --- | --- | --- | --- |
| N50 = 87,672,018, n = 5 |  |  |  |  |
| N60 = 87,672,018, n = 5 |  |  |  |  |
| N70 = 81,620,799, n = 6 |  |  |  |  |
| N80 = 80,330,186, n = 7 |  |  |  |  |
| N90 = 78,333,432, n = 8 |  |  |  |  |
| N100 = 1,000, n = 14,276 | |  |  |  |
| N_count = 22,438,724 |  |  |  |  |
| Gaps = 33,032 |  |  |  |  |
|  |  |  |  |  |
|  |  |  |  |  |
| The 8 pseudomolecules: | Length | Scaffold_id | Percentage in the genome assembly | Total % in the genome assembly |
| 1 | 114,924,005 | Scaffold_5532 | 14.66% | 14.66% |
| 2 | 96,182,003 | Scaffold_9996 | 12.27% | 26.93% |
| 3 | 88,055,906 | Scaffold_15334 | 11.23% | 38.16% |
| 4 | 87,985,974 | Scaffold_10796 | 11.22% | 49.39% |
| 5 | 87,672,018 | Scaffold_8152 | 11.18% | 60.57% |
| 6 | 81,620,799 | Scaffold_3585 | 10.41% | 70.98% |
| 7 | 80,330,186 | Scaffold_10546 | 10.25% | 81.23% |
| 8 | 78,333,432 | Scaffold_10433 | 9.99% | 91.23% |

**Supplementary Table S3.**

| **Summary Genome Information** | |
| --- | --- |
| Sequences in Query Fasta | 15,358 |
| Total Genome Length (bp) | 787,906,105 (765,481,005 excl N/X-runs) |
| GC level | 37.98% |
| Bases masked | 482,392,546 (61.22%) |

| **Class/Family** | **Number of Elements** | **Length Occupied (bp)** | **Percentage of Sequence (%)** |
| --- | --- | --- | --- |
| **SINEs** | 467 | 71,806 | 0.01 |
| **LINEs** | 25,299 | 16,662,662 | 2.11 |
| **LTR elements** | 246,922 | 287,346,678 | 36.47 |
| **DNA elements** | 107,352 | 50,383,707 | 6.39 |
| **Unclassified** | 224,446 | 120,834,671 | 15.34 |
| **Total interspersed repeats** | 604,486 | 475,299,524 | 60.32 |
| **Other (Simple, Small RNA, Satellites, Low Complexity)** | 16,902 | 7,093,022 | 0.90 |

**Supplementary information S4.**

**Supplementary information S5.**

**MVP & MEP Pathway**

**JA biosynthesis pathway**

**Sesquiterpene synthases**

**Supplementary information S6**


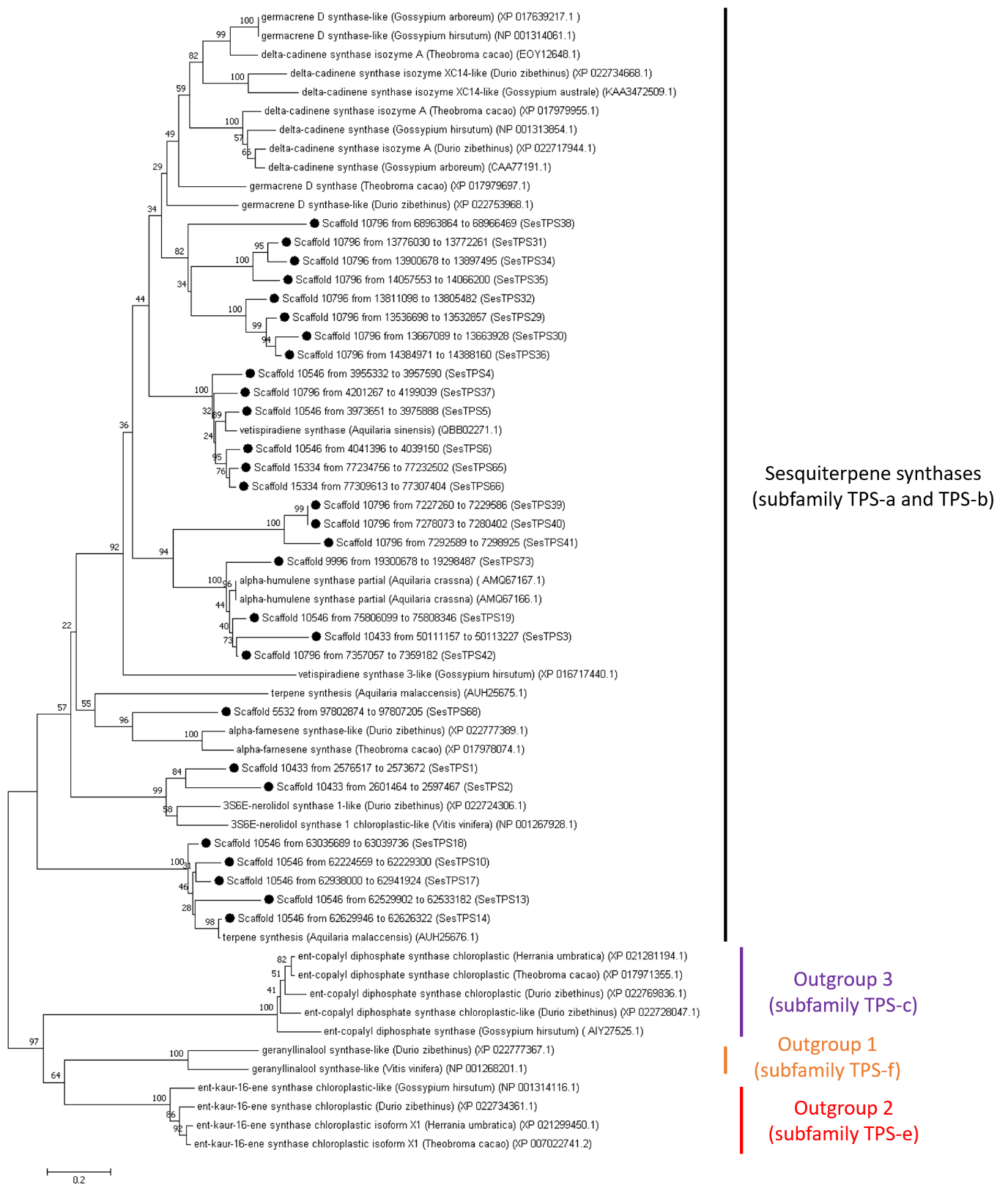


Neighbour-joining tree of sesquiterpene synthases (SesTPSs) constructed based on the alignment of the terpene synthase C terminal domain with 1000 bootstraps. 29 selected full length SesTPSs from our incense tree genome (marked in black) were clustered to available Malvales sesquiterpene genes. Terpene synthase (TPS) genes from subfamily TPS-c (in purple), TPS-e (in red) and TPS-f (in yellow) in Rosids available from the NCBI were selected as outgroups.


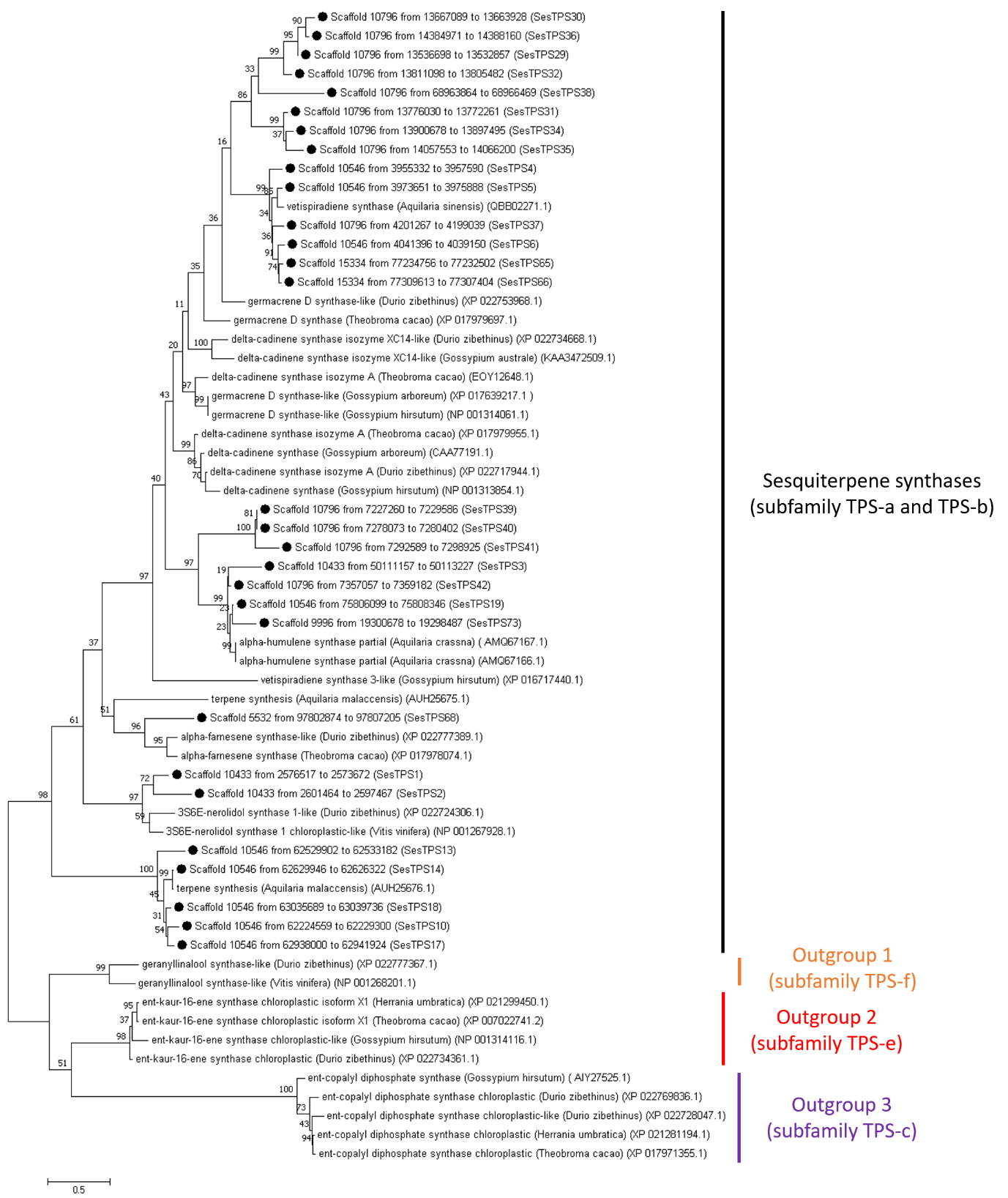


Maximum-likelihood tree of sesquiterpene synthases (SesTPSs) constructed based on the alignment of the terpene synthase C terminal domain with the General Reversible Mitochondrial + Freq. model and 1000 bootstraps. 29 selected full length SesTPSs from our incense tree genome (marked in black) were clustered to available Malvales sesquiterpene genes. Terpene synthase (TPS) genes from subfamily TPS-c (in purple), TPS-e (in red) and TPS-f (in yellow) in Rosids available from the NCBI were selected as outgroups.
